# Soybean-Oil-Body-Substituted Low-Fat Ice Cream with Different Homogenization Pressure, Pasteurization Condition, and Process Sequence: Physicochemical Properties, Texture, and Storage Stability

**DOI:** 10.3390/foods11172560

**Published:** 2022-08-24

**Authors:** Wan Wang, Jinzhe Li, Min Wang, Liya Gu, Zhijing Liu, Cong Xu, Jiage Ma, Lianzhou Jiang, Zhanmei Jiang, Juncai Hou

**Affiliations:** College of Food Science, Northeast Agricultural University, Harbin 150030, China

**Keywords:** soybean oil body ice cream, homogenization, pasteurization, texture, melting properties, storage stability

## Abstract

The purpose of this research was to explore the impacts of different homogenization pressures, pasteurization conditions, and process sequence on the physical and chemical properties of soybean oil body (SOB)-substituted low-fat ice cream as well as the storage stability of SOB-substituted ice cream under these process parameters. With the increase of homogenization pressure (10–30 MPa), the increase of pasteurization temperature (65 °C for 30 min–85 °C for 15 min), and the addition of SOB before homogenization, the overrun and apparent viscosity of ice cream increased significantly, and the particle size, hardness, and melting rate decreased significantly. Thus, frozen dairy products of desired quality and condition could be obtained by optimizing process parameters. In addition, the SOB ice cream showed better storage stability, which was reflected in lower melting rate and hardness and more stable microstructure compared with the full-milk-fat ice cream. This study opened up new ideas for the application of SOB and the development of nutritious and healthy ice cream. Meanwhile, this research supplied a conceptual basis for the processing and quality optimization of SOB ice cream.

## 1. Introduction

Ice cream is frozen foam formed by infusing air during the freezing process [1]. Meanwhile, fat is an important component of ice cream, which plays a major part in maintaining the dryness, structural shape, melting resistance, and taste of ice cream [2]. Therefore, eliminating or decreasing the fat mass in ice cream can lead to various defects in the final quality of the product. However, milk fat is mainly composed of saturated fatty acids, which account for 65–70% of milk fat, and excessive intake of saturated fatty acids increases the risk of nonalcoholic liver disease and obesity [3,4,5]. With the global obesity epidemic, increasing consumer demand to reduce calorie intake, medium- and low-fat dairy products are gaining popularity [6,7]. Therefore, a variety of fat substitutes are now being used in ice cream formulations to lessen the negative impacts of fat reduction or removal on ice cream products.

Soybean oil body (SOB) is an important organelle for storing lipids in soybean. It is a natural oil-in-water emulsion and remains stable under environmental stress, so SOB can be used in a variety of foods (e.g., cream, mayonnaise, ice cream) preparation [8]. SOB is consisted of an outer layer of phospholipid molecules and triglycerides surrounded by intact membrane proteins, similar in structure to milk fat globules. SOB surface proteins are embedded in the interfacial phospholipid layer, giving liposomes unique, extended hydrophobic domains [9,10]. Therefore, the steric hindrance between the SOBs can stabilize the structure of the SOB and play a major part in preventing the aggregation of the SOB [11]. In addition, the SOB contains not only a lower fat content but also a variety of bioactive components, including unsaturated fatty acids, vitamin E, and phytosterols, compared to milk fat globules of equal quality [9]. Therefore, based on its structural characteristics and bioactive activity, SOB can be used as a healthier and more economical fat substitute to reduce consumer intake of total fat and saturated fatty acids.

In a previous study, the low-fat ice cream with SOB substitute for 40% cream had better melting properties, texture, sensory scores, and digestive properties compared to full-cream ice cream [10]. Among them, the fat content of full-cream ice cream and SOB-substituted (40%) ice cream was 5.97 ± 0.04% and 5.63 ± 0.02%, respectively, and the unsaturated fatty acid content was 30.77 ± 0.10% and 44.12 ± 0.36%. It has been confirmed that SOB substitution for cream could significantly decrease the total fat and saturated fatty acid content of ice cream. However, SOB as a milk fat substitute faces the challenge brought by processing conditions, such as homogenization pressure, pasteurization temperature, and process sequence, etc., in the actual production and application process. Homogenization is the key step of raw milk pretreatment, and the strong shear force produced by homogenization will change the conformation of raw milk [12]. Homogenization has been reported to decrease fat globule size and improve the viscoelasticity of dairy raw materials. At the same time, homogenization can change the microstructure, hardness, and rheological properties of ice cream and give new products better properties [13]. In addition, dairy products may constitute a favorable environment for the growth of pathogenic bacteria. Therefore, the pathogenic bacteria in dairy products can be effectively controlled by pasteurization to prolong the shelf life of dairy products [14]. In addition, pasteurization does not affect the size and distribution of fat globules in dairy raw materials but affects the composition and state of protein, which has an important impact on the structure and sensory quality of ice cream [15]. However, there are few reports on the effects of homogenization pressure, pasteurization method, and process sequence on the physicochemical properties, texture, and storage stability of SOB-substituted ice cream. This greatly limits the application and development of SOB as a substitute for cream to prepare low-fat ice cream.

In this research, the SOB substitute for cream to obtain the low-fat ice cream. The effects of different homogenization pressures, pasteurization conditions, and process sequences on the particle size and rheological properties of ice cream mixes as well as on the overrun, melting properties, and texture of ice cream were investigated. In addition, the changes of melting properties, hardness, color properties, and microstructure of SOB ice cream prepared by the optimal process during frozen storage were investigated. This study provides new ideas for the application of SOB and the development of nutritious and healthy ice cream. Meanwhile, this research also provides a theoretical foundation for the processing and quality optimization of SOB ice cream.

## 2. Materials and Methods

### 2.1. Materials

Milk cream with 35% (*w/w* fat was obtained from Qingdao Nestle Co., Ltd. (Qingdao, China). Soybeans were provided by Soybean Institute of Northeast Agricultural University (Harbin, China). Skimmed dried milk was obtained from Inner Mongolia Yili Industrial Group Co., Ltd. (Hohhot, China). All other reagents in this study were of analytical grade.

### 2.2. Extraction of SOB

The SOB was extracted referring to the report of Zhou et al. [16]. Soybeans were washed and soaked in deionized water (25%, *w*/*v*) for 12 h at a constant temperature of 4 °C. The swollen soybeans were ground in pre-cooled deionized water (4 °C) for 2 min by the tissue grinder. The homogenate was filtered through 4 layers of degreased gauze to collect raw soybean milk. The sucrose was added to the raw soybean milk to a final concentration of 20% (20%, *w*/*v*), and mixed rapidly in the ice-water bath. The mixture was centrifuged (8000× *g*, 20 min, 4 °C) to collect SOB cream. The SOB cream was then resuspended in sucrose solution (20%, *w*/*v*) and centrifuged (8000× *g*, 20 min, 4 °C). The above washing step was repeated three times, and the final wash medium did not contain sucrose to collect the SOB. The fat, protein, and moisture content in SOB were determined in previous studies as 25.39 ± 0.28%, 9.27 ± 0.19% and 56.41 ± 0.72%, respectively.

### 2.3. Preparation of SOB Ice Cream

Based on the results of previous studies, low-fat ice cream had better physicochemical properties when SOB was substituted for 40% of milk fat [10]. Thus, the low-fat ice cream with 40% of SOB substituted for cream was used to explore the effect of process parameters on it.

We first mixed 14 g of skim milk powder, 10 g of white sugar, 5 g of egg yolk, 7.2 g of cream, 4.8 g of SOB, and 59 g of water and filter. Among them, the ice cream prepared by replacing the SOB with cream was used as the control group (i.e., the added amount of cream was 12 g). The mixed raw materials were homogenized, pasteurized, and aged (constant temperature of 4 °C for 12 h) to obtain the ice cream mixes. Then, the above mixes were frozen (constant temperature of −5 °C for 30 min) by an ice cream machine (ICM-15S, Jinbangda, Ningbo, China) and hardened (−18 °C, 1 day) in the containers to obtain the ice cream samples by a low-temperature refrigerator (EC5U, Haier, Qingdao, China). The fat, protein, and carbohydrate contents in SOB-substituted ice cream samples were determined in previous studies as 5.63 ± 0.02%, 5.92 ± 0.04%, and 18.48 ± 0.02%, respectively.

When exploring the impact of homogenization pressure on SOB ice cream, the pasteurization condition was determined at 75 °C for 20 min, and the homogenization pressures were selected as 10 MPa, 15 MPa, 20 MPa, 25 MPa, and 30 MPa, respectively, by a high-pressure homogenizer (AH-NANO, Zhengqian Biotechnology Co., Ltd., Shanghai, China).

When exploring the effect of pasteurization conditions on SOB ice cream, the homogenization pressure was determined to be 15 MPa, and the pasteurization conditions were selected as 65 °C for 30 min, 75 °C for 20 min, and 85 °C for 15 min, respectively, by the Cherry-Burrell pasteurizer (EOS-75, Cherry-Burrell Corp., Saint Paul, MN, USA).

When exploring the effect of process sequence on SOB ice cream, the homogenization pressure was determined to be 15 MPa, and the pasteurization condition was 75 °C for 20 min. The SOB added before homogenization and the SOB added after homogenization were named as process 1 and process 2, respectively.

### 2.4. Rheological Properties

The determination of rheological properties was based on the method reported by Jiang et al. [17]. The ice cream mixes (4 °C) were placed on a rheometer plate (MARS40, Thermo, MA, America) to determine the apparent viscosity, flow behavior index (n), and consistency index (K). The shear rate range and the temperature were set as 1–100 s^−1^ and 4 °C, respectively.

### 2.5. Particle Size

The effect of different homogenization pressures, pasteurization conditions, and process sequences on the particle size distribution of ice cream mixes (4 °C) was determined based on the previously reported method [18,19].

### 2.6. Overrun of Ice Cream

The determination of overrun was based on the description of Wang et al. [10]. The same volume of frozen ice cream samples (−18 °C) and of unfrozen ice cream mixes (4 °C) were weighed immediately to record as M_1_ and M_2_, respectively.

Overrun (%) = 100 × (M_1_ − M_2_)/M_2_ (1)

### 2.7. Melting Properties

The determination of melting rate and the first dripping time was based on the method described by Kurt et al. [20]. The ice creams (−18 °C) were placed on a mesh rack at a constant temperature of 37 °C for 1 h to record the time of the first drop. The mass of the ice cream before melting was registered as W_1_. The mass of the melted ice cream was registered as W_2_.

Melting rate (%) = 100 × W_2_/W_1_ (2)

### 2.8. Texture

The texture profile analysis of SOB-substituted low-fat ice cream was determined by the back extrusion method [21]. Low-fat ice cream samples (∅: 40 mm; height: 60 mm) were tested on a texture analyzer (TA-XT Plus, SMATA, Godalming, UK) with a P/50 probe (∅, 50 mm). The pre-test, test, and post-test speed were 1 mm/s; the trigger force was 20 g, the compression strain was 30%, and the time interval between two compressions was 5 s. The texture properties of fresh SOB-substituted low-fat ice cream samples were determined immediately at the temperature of 4 °C after hardening at −18 °C for 24 h.

### 2.9. Sensory Evaluation

To assess consumer acceptance of SOB ice cream with different processing parameters, the appearance, ice crystals, smoothness, chewiness, and acceptability were evaluated. Ice cream samples were prepared before sensory testing, divided into individual test containers, and stored at −20 °C. Sensory scoring of ice cream samples was performed by 10 professionally trained sensory evaluation members, based on the nine-scale hedonic test.

### 2.10. Physicochemical Properties of SOB Ice Cream during Frozen Storage

According to the results of the single-factor test, the SOB ice cream with the SOB added before homogenization, with a homogenization pressure of 20 MPa and a pasteurization condition of 85 °C for 15 min, was selected as the research object to further explore the effect of frozen storage on the SOB ice cream.

SOB ice cream and full-milk-fat ice cream prepared according to the above parameters were stored at a constant temperature of −18 °C for 28 days. The ice creams were sampled weekly to determine melting properties, hardness, color properties, and microstructure during frozen storage.

### 2.11. Color Properties of SOB Ice Cream during Frozen Storage

The determination of color properties of the ice cream samples (−18 °C) were according to the previously reported method [22], including lightness (L*), red-green value (a*), and yellow-blue value (b*), by a colorimeter at the temperature of 4 °C (ZE6000, Nippon Denshoku, Tokyo, Japan).

### 2.12. Microstructure of SOB Ice Cream during Frozen Storage

All the samples were stained according to the previous method [23]. A diluted ice cream sample (2 mL) was stained with 50 µL of Nile blue (0.1%, *w*/*v*) and 40 µL of Nile red (0.1%, *w*/*v*) and reacted in the dark for 0.5 h. After that, 1.5 µL of the reaction solution was blocked on the glass slide. The microstructure of ice cream samples was observed by ultra-high distraction microscopy (Deltavision OMX SR, GE, Boston, MA, USA).

### 2.13. Statistical Analysis

The mean ± standard deviation (*n* = 3) was used to express all date. One-way analysis of variance (ANOVA) was implemented through SPSS software (SPSS 25.0). *p* < 0.05 indicated significant difference with Duncan’s test.

## 3. Results

### 3.1. Steady Shear Rheological Properties

**Figure 1:**
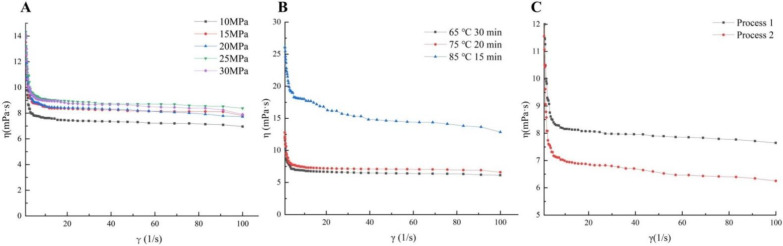
Effects of (**A**) homogenization pressure, (**B**) pasteurization conditions, and (**C**) process sequence on apparent viscosity of soybean oil body ice cream mixes. Process 1 represents soybean oil body added before homogenization, and process 2 represents soybean oil body added after homogenization.

Homogenization is an important factor affecting the tissue state and internal structure of ice cream, which is also beneficial to the improvement of the overrun [24]. The apparent viscosity of SOB ice cream mixes had an significant (*p* < 0.05) increase trend with increasing homogenization pressure (Figure 1A), which may be due to the formation of new fat globule membranes at the oil–water interface of the fat globules under the high-speed shearing action of the homogeneous mechanical force. The newly formed fat globule film was cross-linked with the surrounding proteins so that the proteins were tightly packed at the oil–water interface, thereby increasing the gel strength of the SOB ice cream mixes, resulting in a significant increase in the apparent viscosity [13]. In addition, with the increase of homogenization pressure, the droplet diameter in the aqueous phase decreased significantly (*p* < 0.05), thereby decreasing the speed of droplet motion, which was probably another reason for the increase in apparent viscosity [25]. Similarly, the point of Innocente et al. was also that apparent viscosity of ice cream containing 5% fat content increased with increasing homogenization pressure, being consistent with the viewpoint of this research [13].

In addition, the apparent viscosity of SOB ice cream mixes at the pasteurization condition of 75 °C for 20 min was not significantly different from that at 65 °C for 30 min (*p* > 0.05). However, the apparent viscosity increased significantly (*p* < 0.05) at the pasteurization condition of 85 °C for 15 min (Figure 1B). This might be due to the higher pasteurization temperature denatured whey proteins, and the unfolding of whey proteins exposed thiol or disulfide groups that could further form aggregates between denatured whey proteins through covalent bonds [26,27]. Meanwhile, β-lactoglobulin and α-lactalbumin aggregates would further interact with κ-casein on the surface of casein micelles through disulfide bonds [28]. Therefore, the higher apparent viscosity may be due to increased intermolecular tangles and enhanced intermolecular forces due to protein denaturation caused by pasteurization temperature. Stronger intermolecular forces led to more stable interactions between molecules, which hindered molecular flow and resulted in higher apparent viscosity [26].

Furthermore, the apparent viscosity of the ice cream mixes with SOB added after homogenization was significantly lower than the SOB added before homogenization (Figure 1C). This reason was potentially that the SOB was not homogenized and existed in the form of larger fat globules, which made the stability of the ice cream mixes worse and thus decreased the apparent viscosity [25].

Meanwhile, the ice cream mixes all showed a decreasing trend of apparent viscosity with the increasing shear rate. This state was considered to be shear thinning behavior, which could be attributed to the dissociation of cross-linked macromolecular polymers in ice cream mixes under shear force, and the dissociated molecules were rearranged, decreasing the flow resistance, thus giving rise to a decrease in apparent viscosity [29].

In addition, the effects of different homogenization pressure and pasteurization conditions on the flow behavior index (n) and consistency index (K) of SOB ice cream mixes were presented in Table 1. With increasing homogenization pressure and pasteurization temperature, the K value of SOB ice cream mixes had a significant upward trend, and the n value decreased significantly (*p* < 0.05). Among them, the larger K value showed the higher viscosity, indicating the poor fluidity of ice cream mixes. A smaller n value indicated increased pseudoplastic behavior of ice cream mixes [30]. Furthermore, the n value of the ice cream mixes with SOB added after homogenization was longer, indicating that the mixes were thinner and more fluid, which had a great negative impact on the formation of the ice cream texture. The research cleared that the homogenization pressure of 25 MPa and the pasteurization conditions of 85 °C for 15 min could significantly improve the apparent viscosity and consistency of ice cream mixes. The improvements of rheological properties of ice cream mixes had an influence in the texture of ice cream and also had an impact on overrun and melt resistance of ice cream [20].

### 3.2. Particle Size

**Figure 2:**
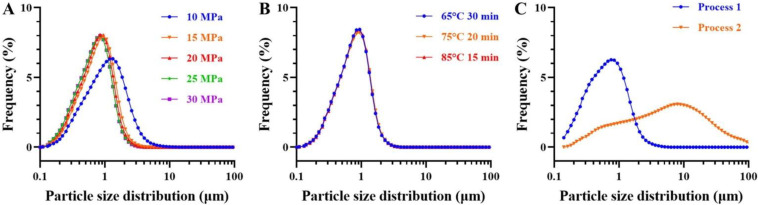
Effects of (**A**) homogenization pressure, (**B**) pasteurization conditions, and (**C**) process sequence on particle distribution of soybean oil body ice cream mixes. Process 1 represents soybean oil body added before homogenization; process 2 represents soybean oil body added after homogenization.

The homogenization pressure had a significant influence in the particle distribution and uniformity of the SOB ice cream mixes (Figure 2A). With the homogenization pressure increasing from 10 to 20 MPa, the mean particle diameter of the SOB ice cream mixes decreased from 1.31 to 0.80 µm. Potentially, the increase in homogenization pressure dispersed the milk fat globules and SOB into smaller fat globules, thereby decreasing the average particle size that is consistent with the particle size change of high-pressure homogenization for milk ice cream with 5% fat content [13]. Generally speaking, the particle size of fat globules varied with the applied homogenization pressure and decreased exponentially [31]. However, when the homogenization pressure was 30 MPa, the D_[4,3]_ of the SOB ice cream mixes was no longer decreased, which was not significantly different from the D_[4,3]_ of the ice cream mixes at 20 and 25 MPa (*p* > 0.05). This might be because even if the homogenization pressure continued to increase, the fat globules that were too small would re-aggregate under the action of the emulsifier so that the average particle size of the ice cream mixes was no longer reduced. In addition, the polydispersity index (PDI) of the ice cream mixes also decreased significantly with increasing homogenization pressure, which indicated that particle distribution of the droplets was more uniform [32].

In addition, there was no significant different between the particle distribution of SOB ice cream mixes prepared with different pasteurization parameters (*p* > 0.05) (Figure 2B and Table 2). The mean particle diameters of the SOB ice cream mixes under the different pasteurization conditions were evenly distributed at 0.94 µm, and the particle distributions were uniform.

The effect of process sequence on particle distribution of SOB ice cream mixes is presented in Figure 2C and Table 2. The mean particle diameter of the ice cream mixes with SOB added before homogenization was at 0.74 µm. Meanwhile, due to the uniform particle distribution of the ice cream mixes and the low degree of aggregation of fat globules, the liquid-dispersion system of ice cream mixes with SOB added before homogenization was relatively statically stable. However, the droplets with SOB added after homogenization would aggregate during the aging process, resulting in an increase in the D_50_, D_[4,3]_, and D_[3,2]_ of the ice cream mixes and a wider particle size distribution [33].

The smaller particle size and PDI meant that the ice cream mixes had better stability [34]. Meanwhile, the food with small particles was easier to maintain structural integrity during oral chewing, thus giving ice cream a smoother taste [35].

### 3.3. Overrun

**Figure 3:**
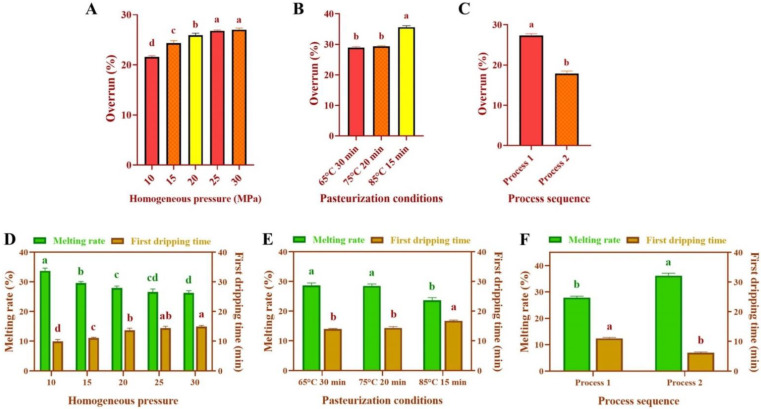
Effects of (**A**,**D**) homogenization pressure, (**B**,**E**) pasteurization conditions, and (**C**,**F**) process sequence on overrun and melt properties of soybean oil body ice cream. Different lowercase letters represent significant differences between treatments of the same indicator (*p* < 0.05). Process 1 represents soybean oil body added before homogenization, and process 2 represents soybean oil body added after homogenization.

The overrun of SOB ice cream increased significantly with increasing homogenization pressure (*p* < 0.05) (Figure 3A). The overrun of ice cream increased from (21.59 ± 0.24)% to (27.01 ± 0.34)% with the increasing homogenization pressure from 10 to 30 MPa.

Moreover, compared with the pasteurization condition of 65 °C for 30 min, the overrun of SOB ice cream under the pasteurization condition of 85 °C for 15 min increased significantly (*p* < 0.05), from (28.89 ± 0.37)% to (35.63 ± 0.49)% (Figure 3B).

Furthermore, the overrun of ice cream with SOB added after homogenization was significantly decreased (*p* < 0.05), in comparison to ice cream with SOB added before homogenization (Figure 3C). The overrun of ice cream with process 1 was (27.33 ± 0.45)%, while the overrun of ice cream with process 2 was only (17.87 ± 0.65)%.

Increased homogenization pressure and pasteurization temperature decreased the droplet size in ice cream mixes and resulted in more uniform particle distribution, which facilitated the entry of air during freezing, thereby increasing the overrun [36]. Meanwhile, the decrease in the particle size of the ice cream mixes indicated that the mixes had better stability, which could maintain the intact shape of the bubbles and prevent the decrease of the overrun of the ice cream caused by the collapse of the bubbles [10]. Meanwhile, the increase in the apparent viscosity of the ice cream mixes owing to the homogenization pressure and pasteurization conditions also made a significant impact on the increase in the overrun of ice cream. This is because the air in the ice cream mixes with higher apparent viscosity will be formed into stable, small air cells during the freezing process, resulting in an increase in the overrun [37]. Meanwhile, the increased apparent viscosity of the ice cream mixes indicated that the proteins in the mixes were cross-linked with the fat to form a stable steric structure, giving rise to a decrease in fluidity. Therefore, the decrease in the fluidity of the ice cream mixes could prevent the collapse of the bubbles due to the change of shape during the freezing process, resulting in the increase of overrun [20]. The increase in overrun could suppress ice crystal collisions and improve melt resistance and foam stability of ice cream during storage [38]. These results were compatible with the description of Biasutti et al. [39] that conventional homogenization could increase the overrun of milk ice cream, which might be related to the influence of processing parameters on the viscosity of ice cream mixes. Meanwhile, the lower apparent viscosity of the ice cream mixes made them difficult to encapsulate the air bubbles and prevented the air bubbles from gathering during the freezing. As a result, less air was infused to the ice cream, resulting in lower overrun [10]. The apparent viscosity of the ice cream mixes with SOB added after homogenization was lower so that the ice cream was filled with less air during the freezing process, thereby decreasing the overrun.

### 3.4. Melting Properties

The melting rate of SOB ice cream had a significant decrease trend with increasing homogenization pressure, while the first drop time of ice cream was significantly prolonged (*p* < 0.05) (Figure 3D). The melting rate of ice cream decreased from (33.76 ± 0.92)% to (26.30 ± 0.74)%, while the first drop time increased from (9.94 ± 0.63) min to (14.92 ± 0.41) min, with the homogenization pressure from 10 MPa increasing to 30 MPa. In accordance with the results of this research, Sert et al. also reported [40] that homogenization (25 MPa) gave rise to a significant reduction in the melting rate and a significant increase in the first drop time of milk ice cream.

In addition, compared with the pasteurization condition of 65 °C for 30 min, the speed of melting of SOB ice cream under the pasteurization condition of 85 °C for 15 min was significantly decreased, while the first drop time was significantly longer (*p* < 0.05) (Figure 3E). When the pasteurization condition was 65 °C for 30 min, the melting rate of ice cream was (28.73 ± 0.77)%, and the first drop time was (13.98 ± 0.21) min. When the pasteurization condition was 85 °C for 15 min, the ice cream melting rate reduced to (23.64 ± 0.93)%, and the first drop time increased to (16.74 ± 0.27) min.

Furthermore, the melting speed of the ice cream with SOB added after homogenization was significantly faster than that with SOB added before homogenization, while the first drop time was just the opposite (*p* < 0.05) (Figure 3F). The first drop time and melting rate of the ice cream with SOB added before homogenization were (12.41 ± 0.34) min and (27.87 ± 0.54)%, respectively. However, the first drop time and melting rate of the ice cream with SOB added after homogenization were and (6.98 ± 0.29) min and (36.22 ± 0.93)%, respectively.

The reason for this might be that the homogenized fat globules were more likely to partially coalesce and form a continuous three-dimensional network during the freezing and whipping process of the ice cream mixes. It had been reported that partial aggregation and fat destabilization of fat globules during freezing are the ideal phenomena for maintaining the structural stability of ice cream and are negatively correlated with the melting rate (r^2^ = −0.963) [41].

Melting was defined by Kalicka et al. as the degree to which an ice cream sample resisted flow when force was applied in the mouth [42]. Therefore, the increase in apparent viscosity of ice cream mixes might be related to the melting resistance. Javidi et al. [43] demonstrated that the high apparent viscosity of ice cream mixes resulted in better melting resistance of ice cream. This might be due to the increasing apparent viscosity of the ice cream mixes, which slowed the migration of water molecules and increased the flow resistance of the mixes, leading to decrease the melting speed and prolong first drop time of the ice cream [43]. In addition, the decreased particle size of the ice cream mixes meant that the network structure of ice cream was more stable, which could also reduce the fluidity of melted ice cream and ensure that the ice cream maintained the original shape during the melting process [44]. In addition, the overrun was closely related to the melt rate. This might be owing to the higher overrun, meaning more air cells in the ice cream. The poor thermal conductivity of air would reduce the transfer of heat energy, thus reducing the melting speed of ice cream [45]. Therefore, the effects of homogenization pressure, pasteurization conditions, and process sequence on rheological properties, particle size, and overrun were all potential factors to decrease the melting rate and prolong the first drop time of SOB ice cream.

### 3.5. Texture

The hardness of ice cream decreased significantly with increasing homogenization pressure (*p* < 0.05) (Table 3). The hardness of ice cream decreased from (4729.48 ± 88.62) g to (4051.69 ± 62.02) g, according to the homogenization pressure increase (10–30 MPa). However, the chewiness and adhesiveness of ice cream had significant increase trends with increasing homogenization pressure (*p* < 0.05). Among them, the adhesiveness increased from (281.08 ± 7.04) g·s to (476.97 ± 3.79) g·s, and the chewiness increased from (279.94 ± 8.33) to (415.73 ± 2.19).

In addition, with the increase of pasteurization temperature, the hardness of SOB ice cream significantly reduced, and the adhesiveness and chewiness of SOB ice cream significantly increased (*p* < 0.05). The hardness of SOB ice cream decreased from (4218.87 ± 45.90) g to (3516.93 ± 75.84) g, the adhesiveness increased from (308.33 ± 4.09) g·s to (458.75 ± 7.43) g·s, and the chewiness increased from (337.84 ± 5.22) to (439.31 ± 9.18).

Furthermore, compared with ice cream with SOB added before homogenization, the ice cream with SOB added after homogenization significantly increased the hardness and chewiness (*p* < 0.05), while the adhesiveness significantly decreased (*p* < 0.05), and conversely, the springiness of ice cream changed with no significant difference (*p* > 0.05).

The decrease in hardness could be associated with the higher overrun of the ice cream. In other words, a higher overrun indicated that less pressure was applied to compress the same volume of samples, and therefore, a lower hardness was perceived at a higher overrun [46]. In addition, the reason for this phenomenon might also be that the hardness of the ice cream was affected by the apparent viscosity of the ice cream mixes [47]. The apparent viscosity of the ice cream mixes affected the rate of ice crystal formation and its size, which in turn affected the hardness of the ice cream [43]. The increase of the homogenization pressure and pasteurization temperature and the addition of SOB before homogenization could significantly increase the apparent viscosity of the ice cream mixes to avoid the formation of more and larger ice crystals, thereby decreasing the hardness of the ice cream [48]. These findings were consonant with the viewpoint by Biasutti et al. [39], who reported that although no clear relationship between hardness and homogenization pressure was observed, this behavior was potentially related to the effect of processing parameters (homogenization pressure or pasteurization) on the overrun and apparent viscosity of the ice cream. At the same time, the increase of homogenization pressure and pasteurization temperature and the addition of SOB before homogenization could enhance the stabilization of ice cream by decreasing the droplet diameter of ice cream mixes. The improved stability increased the recovery rate of the ice cream after deformation, indicating that both the yield stress and material stiffness parameters of the ice cream were increased. Thus, the ice cream samples showed higher adhesiveness, springiness, and chewiness [20]. The increasing adhesiveness and the decreasing ice crystal size would give the ice cream a smoother mouthfeel [48]. In addition, the chewiness and springiness were positively correlated with the residence time of ice cream in the oral cavity; thus, the increase of springiness and chewiness could improve the degree of consumer enjoyment of soy and milk flavors [49]. The results were consistent with the result that quince seed improved the texture of ice cream by increasing the apparent viscosity [20].

### 3.6. Sensory Properties

**Figure 4:**
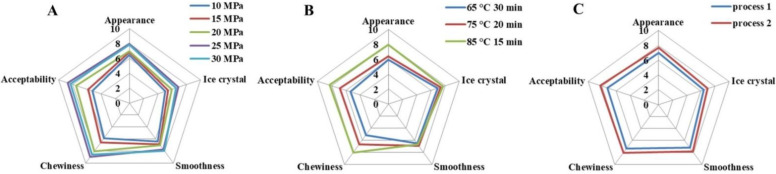
Effects of (**A**) homogenization pressure, (**B**) pasteurization conditions, and (**C**) process sequence on sensory properties of soybean oil body ice cream. Process 1 represents soybean oil body added before homogenization, and process 2 represents soybean oil body added after homogenization.

The sensory evaluation results of 10 professionally trained sensory evaluation panels are shown in Figure 4. The sensory panels were asked to rate the ice cream samples based on appearance, ice crystals, smoothness, chewiness, and acceptability based on the nine-scale hedonic test. When the homogenization pressure was 25 MPa and 30 MPa, the appearance scores of SOB ice cream were 7.94 ± 1.03 and 7.83 ± 0.65, the ice crystal scores were 6.88 ± 1.12 and 6.54 ± 1.32, the smoothness scores were 7.71 ± 0.85 and 7.95 ± 1.32, chewiness scores were 8.97 ± 1.25 and 8.57 ± 0.84, and acceptability scores were 8.72 ± 0.75 and 8.28 ± 0.92, respectively, which were higher than other SOB ice creams. When pasteurization condition was 85 °C for 15 min, the appearance, ice crystals, chewiness, and acceptability of SOB ice cream were 7.94 ± 1.21, 7.72 ± 0.86, 8.05 ± 1.43, and 8.32 ± 0.95, respectively, which were significantly higher than those of pasteurization conditions at 75 °C for 20 min and 65 °C for 30 min. However, the difference between smoothness scores was not significant. In addition, ice cream with SOB added before homogenization had better appearance, ice crystals, smoothness, chewiness, and acceptability scores compared to ice cream with SOB added after homogenization.

As described by members of the sensory assessment panel, the SOB ice cream with homogenization pressures of 25 and 30 MPa and SOB added before homogenization had smooth texture and uniform melting, which was in keeping with the texture results. This might be owing to the decrease in particle size and PDI of ice cream with the change of processing parameters, which indicated that the uniform dispersion of droplets in ice cream was easy to melt in the oral cavity. Additionally, the smoothness indicated that proper processing parameters could eliminate the roughness of SOB ice cream, meaning less ice crystals perceivable in the ice cream [43], which was consistent with the sensory scores of ice crystals as well as the firmness of ice cream. Furthermore, according to the statement of the sensory evaluation panel, the ice cream treated with the homogenization pressure of 25 and 30 MPa, pasteurization conditions of 85 °C for 15 min, and SOB added before homogenization was chewier compared to other groups of ice creams. Usually, during chewing, highly elastic foods undergo reversible deformation, leading to an increase in the number of chews, which is thought to be positively correlated with chewiness. [49]. Therefore, proper processing parameters could increase the chewiness of ice cream. The increased residence time of the ice cream in the oral cavity contributed to this, enhancing the consumer’s oral perception of the SOB ice cream flavor. In conclusion, ice cream prepared with homogenization pressures of 25 and 30 MPa, pasteurization conditions of 85 °C for 15 min, and SOB addition before homogenization had the best overall acceptability to consumers.

### 3.7. Melting Properties of SOB Ice Cream during Storage

**Figure 5:**
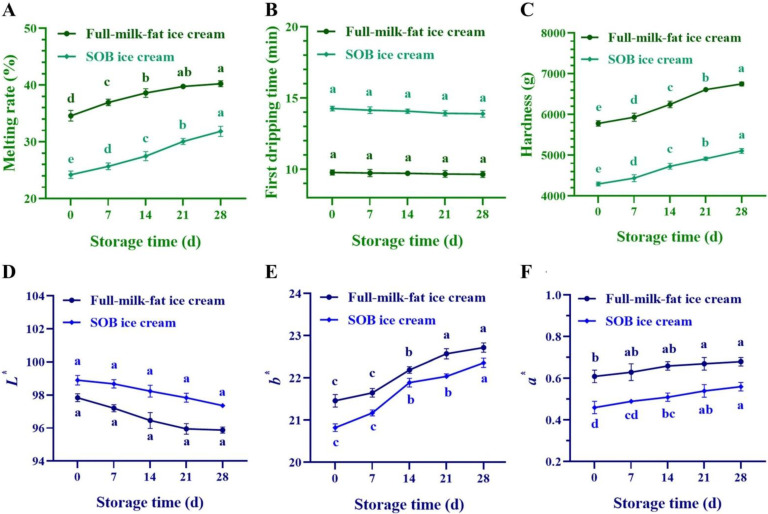
Effects of storage time on (**A**) melting rate, (**B**) first dropping time, (**C**) hardness, (**D**) lightness, (**E**) red-green value, and (**F**) yellow-blue value of SOB ice cream and full-cream ice cream. Different lowercase letters represent significant differences between the same ice cream under different storage times (*p* < 0.05).

The melting properties of the SOB ice cream during storage were presented in Figure 5A. The melting speed of SOB ice cream and full-cream ice cream increased significantly (*p* < 0.05) with the prolongation of storage time. The melting rates of SOB ice cream and full-milk-fat ice cream were (24.21 ± 0.65)% and (34.59 ± 0.94)% in the initial storage period and increased to (31.85 ± 0.88)% and (40.24 ± 0.53)%, respectively, after 28 days of frozen storage. However, there was no significant difference (*p* > 0.05) in the first drop time between SOB ice cream and full-milk-fat ice cream during storage (Figure 5B), which was analogous to the results reported by Guven et al. [50].

The reason for the increased melting rate might be the recrystallization of ice cream during ice cream storage. Recrystallization, also known as ripening or coarsening, would change the shape and size of ice crystals [51], wherein the larger the ice crystal volume, the faster the ice cream melted [52]. Meanwhile, the melting rate of SOB ice cream was lower than that of full-cream ice cream during storage, which was probably owing to the higher overrun of SOB ice cream. Heat is generally transferred from the outside to the inside of the ice cream during the melting process, and higher overrun of the ice cream would decrease the heat transfer rate owing to the low thermal conductivity of the air [53]. Furthermore, the melting rate of ice cream was related to the degree of coalescence of the droplets and the formation of fat–protein polymers [44]. In previous studies, it was found that the SOB-substituted ice cream mixes had a smaller droplet diameter and higher structural stability, which had an important hindering effect on the flow of water molecules, thereby decreasing the melting speed of the SOB ice cream [44].

### 3.8. Hardness of SOB Ice Cream during Storage

The hardness of both ice creams increased significantly with prolonged storage time (*p* < 0.05) (Figure 5C). The hardness of SOB ice cream and full-milk-fat ice cream were (4294.08 ± 46.41) g and (5780.42 ± 66.79) g in the initial storage period and increased to (5107.12 ± 57.40) g and (6750.41 ± 49.38) g after 28 days of frozen storage, and a similar trend was also observed by Pon et al. [47]. This might be due to the fact that during long-term refrigerated storage, the water or water vapor surrounding the ice crystal moved towards the ice crystal, attaching to and freezing on it, resulting in the fine ice crystals gradually merging and growing into larger ice crystals, which was positively correlated with the hardness of the ice cream [52]. Furthermore, unstable ice cream mixes could cause fat globules to destabilize and aggregate during the freezing process, and droplet aggregation might increase the hardness of the ice cream. Because the SOB ice cream mixes had higher stability than the full-cream ice cream, the SOB could maintain its integrity during the freezing process and avoid the aggregation of fat globules due to rupture, thereby decreasing the hardness of the ice cream [52].

### 3.9. Color Properties of SOB Ice Cream during Storage

The color properties of SOB ice cream with different storage times are shown in Figure 5D–F. The effect of storage time on the L* of ice cream was not significantly different (*p* > 0.05), but the a* and b* values increased significantly (*p* < 0.05). On the 28th day of storage, the a* values of SOB ice cream and full-milk-fat ice cream increased from 0.46 ± 0.03 and 0.61 ± 0.03 to 0.56 ± 0.02 and 0.68 ± 0.02, respectively, and the b* values increased from 20.82 ± 0.09 and 21.46 ± 0.15 to 22.36 ± 0.11 and 22.72 ± 0.11, respectively. In this study, the differences in color properties might be owing to changes in the air cells structure, protein, and fat globule states of ice cream during storage, which affected the light-reflecting properties of ice cream [53].

### 3.10. Microstructure of SOB Ice Cream during Storage

**Figure 6:**
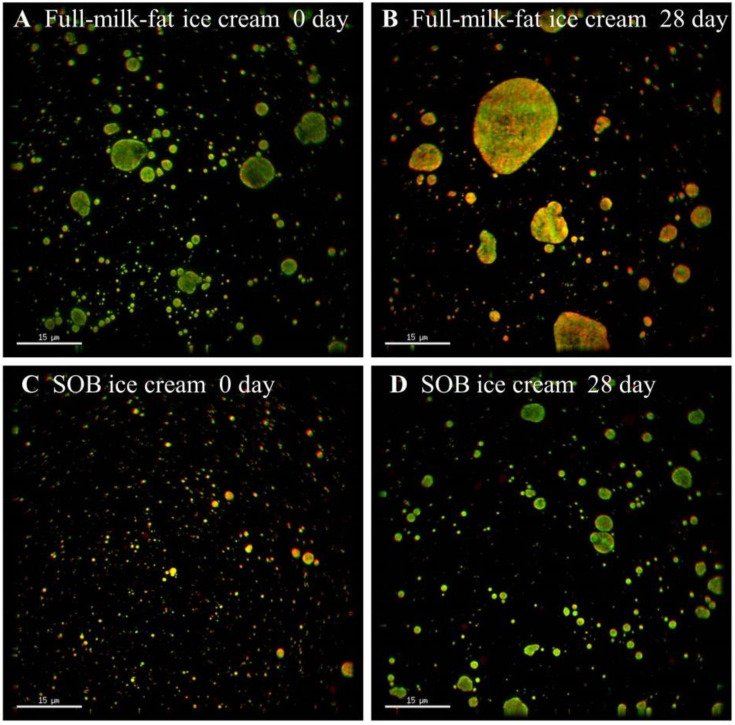
Effects of storage time on the microstructure of SOB ice cream and full-cream ice cream.

The microstructure of fat and protein was collected using ultra high-resolution microscope (fat in green and protein in red). Compared with the initial stage of storage, the droplet size in both the SOB ice cream and the full-milk fat ice cream after 28 days of frozen storage increased significantly (Figure 6). Due to the prolonged storage time of ice cream at low temperature, the fat crystals in the fat globules grew and protruded, destroying the fat globule membrane, and the ruptured fat globules re-aggregated, resulting in larger droplets in the ice cream [6]. However, the droplet aggregation of SOB ice cream was significantly lower than that of full-milk-fat ice cream, indicating that SOB ice cream had better storage stability. This was because the particle size distribution of milk fat globules was relatively broad so that fat crystals protruded from the surface of the fat globules and damaged the milk fat globule membranes when stored at low temperature, resulting in the aggregation of droplets [54]. However, the polarity of phospholipids and the charge of liposome surface proteins increased the mutual repulsion between SOBs to prevent aggregation, so SOBs maintained their physical stability even under processing conditions [55]. Therefore, SOB ice cream had better storage stability than full-cream ice cream, as a consequence of the structural difference between SOB and milk fat globules.

## 4. Conclusions

This study provides a new perspective on the effects of homogenization pressure, pasteurization conditions, and process sequence on the physicochemical properties of SOB ice cream. The study found that the homogenization pressure of 25 or 30 MPa, the pasteurization conditions of 85 °C for 15 min and SOB added before homogenization were the best conditions for SOB ice cream with the best acceptability. Meanwhile, based on the above processing parameters, the apparent viscosity, overrun, and melting properties of ice cream could be significantly improved, and the particle size and hardness of ice cream could be significantly decreased, which were crucial for improving the taste and quality of ice cream. In addition, contrasted with full-cream ice cream, SOB ice cream had better storage stability, which had positive significance for the transportation and sales of frozen dairy products. This research provides a theoretical foundation for the selection of process parameters for frozen dairy products. Meanwhile, this research provides new ideas for the utilization of SOB in food and the development of nutritious and healthy ice cream.

## Figures and Tables

**Table 1:** Effects of homogenization pressure, pasteurization conditions and process sequence on steady shear rheological properties of soybean oil body ice cream mixes.

Factors	Levels	K (Pa·s^n^)	n	R^2^
Homogeneous pressure (MPa)	10	0.28 ± 0.02 ^d^	0.61 ± 0.02 ^a^	0.9357
	15	0.32 ± 0.01 ^c^	0.58 ± 0.01 ^ab^	0.9471
	20	0.33 ± 0.01 ^bc^	0.57 ± 0.01 ^bc^	0.9452
	25	0.38 ± 0.02 ^a^	0.51 ± 0.02 ^d^	0.9427
	30	0.35 ± 0.01 ^b^	0.54 ± 0.02 ^cd^	0.9475
Pasteurization conditions	65 °C, 30 min	0.30 ± 0.01 ^c^	0.58 ± 0.02 ^a^	0.9191
	75 °C, 20 min	0.33 ± 0.02 ^b^	0.55 ± 0.01 ^b^	0.9096
	85 °C, 15 min	0.47 ± 0.01 ^a^	0.49 ± 0.03 ^c^	0.9372
Process sequence	Process 1	0.32 ± 0.01 ^a^	0.58 ± 0.01 ^c^	0.9344
	Process 2	0.20 ± 0.02 ^c^	0.66 ± 0.02 ^a^	0.9207

Different lowercase letters indicate that there were significant differences between treatments of the same indicator (*p* < 0.05); process 1: soybean oil body added before homogenization; process 2: soybean oil body added after homogenization.

**Table 2:** Effects of homogenization pressure, pasteurization conditions, and process sequence on particle size of soybean oil body ice cream mixes.

Factors	Levels	D_50_ (µm)	D_[4,3]_ (µm)	D_[3,2]_ (µm)	PDI
Homogeneous pressure (MPa)	10	1.03 ± 0.02 ^a^	1.16 ± 0.02 ^a^	0.77 ± 0.01 ^a^	0.86 ± 0.02 ^a^
	15	0.80 ± 0.01 ^b^	0.97 ± 0.01 ^b^	0.60 ± 0.01 ^b^	0.61 ± 0.01 ^b^
	20	0.78 ± 0.03 ^c^	0.85 ± 0.03 ^c^	0.61 ± 0.02 ^b^	0.57 ± 0.00 ^c^
	25	0.73 ± 0.01 ^d^	0.80 ± 0.01 ^d^	0.57 ± 0.00 ^c^	0.55 ± 0.01 ^d^
	30	0.73 ± 0.02 ^d^	0.82 ± 0.02 ^cd^	0.57 ± 0.01 ^c^	0.56 ± 0.01 ^cd^
Pasteurization conditions	65 °C, 30 min	0.81 ± 0.02 ^a^	0.87 ± 0.02 ^a^	0.64 ± 0.01 ^a^	0.60 ± 0.01 ^a^
	75 °C, 20 min	0.80 ± 0.01 ^a^	0.87 ± 0.01 ^a^	0.64 ± 0.00 ^a^	0.61 ± 0.01 ^a^
	85 °C, 15 min	0.81 ± 0.02 ^a^	0.88 ± 0.02 ^a^	0.65 ± 0.02 ^a^	0.61 ± 0.01 ^a^
Process sequence	Process 1	0.68 ± 0.01 ^c^	0.79 ± 0.01 ^c^	0.56 ± 0.01 ^c^	0.61 ± 0.01 ^c^
	Process 2	4.08 ± 0.43 ^a^	8.64 ± 0.51 ^a^	1.38 ± 0.08 ^a^	1.59 ± 0.03 ^a^

Different lowercase letters indicate that there were significant differences between treatments of the same indicator (*p* < 0.05); process 1: soybean oil body added before homogenization; process 2: soybean oil body added after homogenization.

**Table 3:** Effects of homogenization pressure, pasteurization conditions, and process sequence on the texture of soybean oil body ice cream.

Factors	Levels	Hardness (g)	Adhesiveness (g·s)	Springiness	Chewiness
Homogeneous pressure (MPa)	10	4729.48 ± 88.62 ^a^	281.08 ± 7.04 ^e^	0.79 ± 0.00 ^b^	279.94 ± 8.33 ^e^
	15	4490.53 ± 96.28 ^b^	310.21 ± 6.72 ^d^	0.81 ± 0.01 ^a^	346.46 ± 6.96 ^d^
	20	4266.15 ± 54.03 ^c^	329.00 ± 5.94 ^c^	0.82 ± 0.00 ^a^	381.28 ± 3.76 ^c^
	25	4184.76 ± 81.65 ^cd^	450.70 ± 10.70 ^b^	0.83 ± 0.01 ^a^	406.99 ± 4.02 ^b^
	30	4051.69 ± 62.02 ^d^	476.97 ± 3.79 ^a^	0.83 ± 0.02 ^a^	415.73 ± 2.19 ^a^
Pasteurization conditions	65 °C, 30 min	4218.87 ± 45.90 ^a^	308.33 ± 4.09 ^c^	0.80 ± 0.01 ^b^	337.84 ± 5.22 ^c^
	75 °C, 20 min	4041.02 ± 36.83 ^b^	316.29 ± 6.72 ^b^	0.82 ± 0.02 ^b^	346.46 ± 6.96 ^b^
	85 °C, 15 min	3516.93 ± 75.84 ^c^	458.75 ± 7.43 ^a^	0.89 ± 0.02 ^a^	439.31 ± 9.18 ^a^
Process sequence	Process 1	4340.50 ± 92.28 ^b^	310.21 ± 6.72 ^a^	0.80 ± 0.01 ^a^	346.46 ± 6.96 ^b^
	Process 2	6849.85 ± 149.10 ^a^	289.34 ± 12.17 ^c^	0.81 ± 0.01 ^a^	369.20 ± 8.03 ^a^

Different lowercase letters indicate that there were significant differences between treatments of the same indicator (*p* < 0.05); process 1: soybean oil body added before homogenization; process 2: soybean oil body added after homogenization.

## Data Availability

Data are contained within the article.
